# Molecular-Assisted Distinctness and Uniformity Testing Using SLAF-Sequencing Approach in Soybean

**DOI:** 10.3390/genes11020175

**Published:** 2020-02-06

**Authors:** Shengrui Zhang, Bin Li, Ying Chen, Abdulwahab S. Shaibu, Hongkun Zheng, Junming Sun

**Affiliations:** 1 The National Engineering Laboratory for Crop Molecular Breeding, MARA Key Laboratory of Soybean Biology (Beijing), Institute of Crop Sciences, Chinese Academy of Agricultural Sciences, 12 Zhongguancun South Street, Beijing 100081, China; Zhangshengrui@caas.cn (S.Z.); libin02@caas.cn (B.L.); chenying@ystwt.com (Y.C.); asshuaibu.agr@buk.edu.ng (A.S.S.); 2 Biomarker Technologies Corporation, Beijing 101300, China; zhenghk@biomarker.com.cn

**Keywords:** soybean (*Glycine max* (L.) Merrill), SSR, SNP, SLAF-seq, fingerprint, cultivar identification

## Abstract

Distinctness, uniformity and stability (DUS) testing of cultivars through morphological descriptors is an important and compulsory part of soybean breeding. Molecular markers are usually more effective and accurate in describing the genetic features for the identification and purity assessment of cultivars. In the present study, we assessed the distinctness and uniformity of five soybean cultivars using both single nucleotide polymorphism (SNP) markers developed by specific-locus amplified fragment sequencing (SLAF-seq) technology, and simple sequence repeat (SSR) markers. The phylogenetic tree and principal component analysis (PCA) from both the SLAF-seq and SSR methods showed a clear distinction among cultivars Zhonghuang 18, Zhonghuang 68 and Zhonghuang 35, while no clear distinction was observed between cultivars Zhonghuang 13 and Hedou 13. Using the SLAF-seq method, we determined the proportion of homozygous loci for the five soybean cultivars. The heterozygosity of each individual plant was estimated for the assessment of cultivar purity and the purity levels of the five soybean cultivars ranged from 91.89% to 93.96%. To further validate the applicability of the SLAF-seq approach for distinctness testing, we used the SNP information of 150 soybean cultivars with different origins. The cultivars were also distinguished clearly. Taken together, SLAF-seq can be used as an accurate and reliable method in the assessment of the distinctness and uniformity of soybean cultivars.

## 1. Introduction

Plant varietal identification and purity assessment play important roles in breeding, registration and trade processes, and they depend mainly on two strategies based on morphological descriptors or molecular markers [1]. Morphological descriptors including plant height, leaf shape, flower color and maturity are traditionally used to distinguish and classify plant species or cultivars [2]. Cultivar registration and protection require distinctness, uniformity and stability (DUS) testing based on morphological features [3]. However, the current DUS testing has some limitations in distinguishing cultivars. For instance, the commercial cultivars are usually bred using narrow elite parent backgrounds, which leads to high similarity in morphological descriptors and thereby results in difficulty in cultivar identification. Furthermore, for the case of maize in China, there are 6291 registered cultivars as of 2013, and they are all required to be evaluated alongside new candidate varieties for accurate DUS assessment [4]. The time-, labor-consumption and sensitivity to environmental changes involved in DUS testing make it vague in distinguishing cultivars [5]. Furthermore, this process of DUS testing using morphological descriptor is mostly restricted to a few traits which are easily influenced by environmental factors [6]. These disadvantages seriously affect the accuracy and efficiency of cultivar identification and may lead to the possibility of infringing or fake varieties passing the DUS testing in cultivar registration, causing economic losses to breeders. In addition to DUS testing based on morphological descriptors, more reliable methods should be applied to identify varieties with similar morphological features in DUS testing or infringement lawsuits. 

Owing to these reasons, the International Union for the Protection of New Varieties of Plants (UPOV), an intergovernmental organization whose system of plant variety protection is intended to encourage innovation in the field of plant breeding [7], has called for the adoption of a DNA-based system that will enable examiners to deploy trait-specific DNA markers in DUS testing [6]. Genetic features have been considered a remarkable improvement for the identification of cultivars in DUS testing [8,9,10,11]. Recently, molecular marker approaches appeared to define the genome variations of different cultivars and have become more important and popular in cultivar identification and genetic map construction [12]. Molecular markers including restriction fragment length polymorphisms (RFLP) [13], random amplified polymorphic DNA (RAPD) [14,15,16], simple sequence repeat (SSR) [17,18], inter-simple sequence repeat (ISSR) [19], and single nucleotide polymorphism (SNP) [20,21], were applied to genetic diversity analysis and genetic mapping. In DUS testing, amplified fragment length polymorphisms (AFLP) and SSR are the most commonly used [6]. SSR markers have the advantage of being highly polymorphic and originate from defined chromosomal locations. Recently, SNP markers have been used in distinctness testing of maize and alfalfa varieties. Compared to SSR markers, bi-allelic SNP markers are more abundant in plant genomes, making them the choice of markers for DNA fingerprinting in modern plant breeding and plant research programs. Additionally, compared with mutable markers, such as microsatellites, SNPs have a low rate of recurrent mutation, making them stable indicators [22]. During the period of Sanger sequencing, the calling of SNPs depended on PCR and Sanger sequencing created low-throughput fingerprint for varieties. With the advantages of the next generation sequencing (NGS) technologies in this decade, high-throughput detection of SNPs is highly accelerated [23,24]. 

Although dramatic falls have occurred in recent decades, the sequencing cost remains an important constraint in many cases [25]. To balance the cost and depth of sequencing, reduced-representation genome sequencing (RRGS) technology was developed [23]. This approach first creates a reduced representation library (RRL) by digesting genomic DNA with a specific restriction enzyme. Subsequently, the RRL is sequenced for SNP calling through NGS technologies. It ensures sufficient accuracy for SNP calling due to more coverage for each SNP when compared to whole-genome resequencing [23]. Specific-locus amplified fragment sequencing (SLAF-seq) is one extensively used RRGS technique, and can generate more than 100,000 tags in each experiment. It ensures the accuracy of the tags by increasing the depth of the tags. Recently, SLAF-seq has been used in QTL mapping [26], genome-wide association analysis [27], and bulk segregant analysis [28]. 

Soybean [*Glycine max* (L.) Merrill] is one of the most widely grown crops for oil, plant protein and other numerous uses. The world production of soybean as of 2017 was 352 million tons [29]. However, not all cultivars produced the same average yield due to genetic variability. Therefore, DUS testing is important for guiding and protecting breeders. Purity contributes to the retention of varietal characteristics, and a positive correlation between varietal purity and yield has been reported. In recent decades, varietal identification and purity assessment based on molecular markers [25,26,27,28,29,30,31] usually had some limitations, such as low-density and low-throughput. This has made DUS testing using molecular markers extraordinarily time-consuming and labor-intensive. Therefore, a more reliable and accurate approach is needed. The ever increasing use of NGS technologies has renewed the focus of researchers on the detailed investigation of molecular-based or molecular-assisted DUS testing in a timelier manner [6]. The DNA-based markers can be used to supplement, or even ultimately replace, existing morphological and protein-based approaches. The UPOV Working Group on Biochemical and Molecular Techniques and DNA Profiling, in particular, have explored such options and proposed two models: the first is using characteristic-specific molecular markers, while the second is combining phenotypic and molecular distances in the management of variety collections [30]. Nowadays, DUS testing based on morphological descriptors is compulsory; however, it may have difficulty distinguishing similar cultivars. Hence, adopting the first model, we developed an accurate and reliable approach to complement the morphological method of DUS testing in soybean using SLAF-seq technology.

## 2. Materials and Methods 

### 2.1. Plant Materials

Soybean cultivars including *cv.* Zhonghuang 13 (ZH13), Zhonghuang 35 (ZH35), Zhonghuang 18 (ZH18), Zhonghuang 68 (ZH68) and Hedou 13 (HD13) were used for the assessment of cultivar uniformity in this study (Table 1). The *cv.* ZH13, ZH35, ZH18 and ZH68 were released by Soybean High-yield and Quality Breeding Research Group in the Institute of Crop Sciences (ICS), Chinese Academy of Agricultural Sciences (CAAS). Cultivar ZH18 is one of the parents for *cv.* ZH68. Cultivar ZH13 is a leading soybean cultivar in the Huanghuaihai valley region of China, while *cv.* ZH 35 is one of the highest yielding soybean cultivars, which had the highest yield record of 6.32 tons per hectare in 2012 in China. Cultivar HD13 was developed by the Heze Academy of Agricultural Science, Shandong Province. The soybean seeds of each cultivar were produced by our research group except *cv.* HD13, which was sourced from a local market in China. Twenty random soybean healthy seeds of each cultivar were grown in vermiculite under a constant temperature of 25 °C and 16 hours of illumination with LED lights at 300 μmol m^−2^ s^−1^. After two weeks, the representative leaf samples from the individual plants were harvested and persevered in liquid nitrogen for genomic DNA extraction.

Furthermore, The dataset of SNPs called by SLAF-seq was obtained from the additional file 2 of the published study on the GWAS of resistance to cyst nematode in soybean [6]. The dataset consists of 36,976 SNPs from 440 soybean varieties and we selected 150 cultivars randomly from 440 cultivars for the construction of phylogenic trees. The 150 cultivars contain 118 Chinese cultivars which originated from three main soybean cultivation regions including Northern Region (NR), Huanghuaihai Region (HR), and Southern Region (SR), 23 cultivars from North America, four from Europe, two from Japan, two from Russia and one from Nigeria.

### 2.2. DNA Extraction, SLAF Library Construction and Sequencing

The genomic DNA extraction was performed using DNase secure Plant Kit (Catalog Number: DP320, TIANGEN Biotech (Beijing) Co., Ltd., Bejing, China). The procedure of SLAF-seq described by Sun et al. [31] was used with some modifications. Briefly, a pilot experiment was performed to evaluate the number, distribution and repetition of the restriction fragment. Genomic DNA was incubated with *Hae*III (New England Biolabs (NEB), Ipswich, USA), T4 ligase (NEB), ATP (NEB) and *Hae*III adaptors at 37 °C for 2 h after which the reaction was heat-inactivated at 65 °C for 15 min. The fragments were digested with *EcoR*I at 37 °C for 2 h and the barcode1 was added to the fragments by PCR using *Hae*III primers. The products were purified by E.Z.N.A.® Cycle Pure Kit (Omega Bio-tek, Norcross, GE, USA) and then pooled in 24 samples. The pooled samples were incubated with *Hae*III, T4-ligase, ATP and Solexa adapter at 37 °C for 2 h and then purified using a Quick Spin column (Qiagen, Hilden, Germany). Fragments of 300–360 bp (with indices and adaptors) were separated by 2% agarose gel and collected by a Gel Extraction Kit (Qiagen, Hilden, Germany). The barcode2 was then added using the Solexa amplification primer mix by Phusion Master Mix (NEB). Fragments of 300–360 bp were gel-purified for sequencing. Pair-end sequencing was done using the Illumina high-throughput sequencing platform (Illumina, San Diego, USA).

### 2.3. SSR Detection

The primers for the SSR markers were synthesized according to the sequences reported by Guan et al. [32] and the forward primer of each SSR was labeled with 6-FAM or HEX fluorescent dyes (Table S1). The PCR reaction mix was prepared as follows: 20 µL reaction solution containing 30 ng of total genomic DNA, 2 pM of forward and reverse primers, 1 µL dNTP, and 0.25 µL Taq polymerase (Transgen, Beijing, China). The PCR was performed under the conditions of 95 °C for 3 min and subsequent 35 cycles of 94 °C for 30 s, 55 °C for 30 s, and 72 °C for 30 s, using a Bio-rad C1000 Touch Thermocycler (Bio-rad, Hercules, USA). Of the diluted PCR product (1/30 times), 0.3 µl containing 500 bp ROX internal marker was resolved by PAGE on an ABI 3730XL DNA sequencer (Perkin Elmer, Waltham, USA). The fragment sizes were automatically analyzed using Genemapper V3.7 software (Thermo Fisher Scientific, Waltham, MA, USA).

### 2.4. SLAF-seq Data Grouping and Genotyping

Grouping and genotyping of SLAF-seq data were carried out by the procedures described by Sun et al. [31]. Briefly, the high-quality reads (error chance < 0.01%, QC30) were mapped onto the soybean genome (Glyma.Wm82.a2) using BWA software [33,34]. The reads with over 90% identity were considered as the identical SLAF markers and grouped in one SLAF locus. The Genome Analysis Toolkit [35] and SAMtools [36,37] were applied for SNPs discovery and the common SNPs were used for subsequent analysis. Alleles were defined in each SLAF using the minor allele frequency (MAF) evaluation. The SNPs with MAF < 0.05 and integrity > 0.8 were collected and used for subsequent analysis.

### 2.5. Data Analysis

The phylogenic trees from the SNP and SSR data were constructed by the neighbor-joining method with bootstrap value 500 using Mega 10 software [38,39,40] and used for assessment of distinctness and uniformity of cultivars. The visualization and annotation of the phylogenic trees were carried on by ggtree package in R project [41,42]. Principal component analysis (PCA) and kinship analysis were further conducted to assess the cultivar distinctness and cultivar specific SNPs. The PCA and kinship analyses using SNPs were performed using the GAPIT package in R project [43]. The PCA for SSR data was performed using the PCA function in R project.

### 2.6. Assessment of Purity

The ratio of the homozygous loci of each individual plant was analyzed and considered as the purity of the plants. The purity of a cultivar was the average of all the individual plants of these cultivars.

## 3. Results

### 3.1. Assessment of Distinctness and Uniformity of Soybean Cultivars Based on SLAF-seq

**Figure 1:**
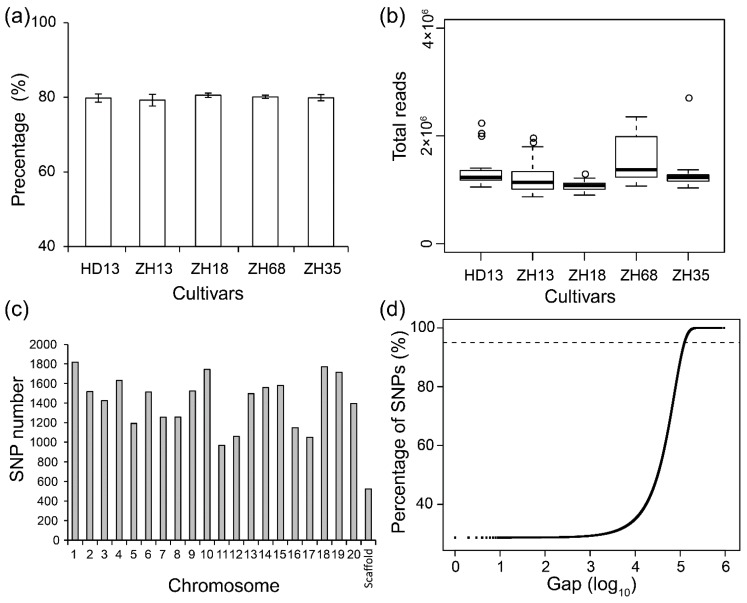
The basic information about specific-locus amplified fragment sequencing (SLAF-seq) results in five soybean cultivars. (**a**) The ratio of high-quality reads (QC30) in SLAF-seq for five soybean cultivars; (**b**) The total read number of SLAF-seq; (**c**) The single nucleotide polymorphism (SNP) number in individual chromosomes; (**d**) The distribution of the gaps between every two adjacent SNPs. The dashed line indicates the percentage of SNPs with gaps of less than 150 kb.

In order to assess the uniformity of soybean cultivars, multiple plants of five cultivars were subjected to SLAF-seq. A total of 9.89 Gb data consisting of 53,078,875 pair-end reads were generated with a high-quality ratio (0.1% chance of error, QC30) of above 80.0% (Figure 1a). The average number of reads from each individual soybean plant was 1.29 million (Figure 1b). The average GC content (42.27%) of all the cultivars was not significantly different. Over 90% of the high-quality reads were mapped to the reference soybean genome (Wm82 a2v1) by BWA software and this covered 5.98% of the genome. In total, 163,134 SNPs were called by GATK and SAMtools from the five cultivars and the SNPs in each plant ranged from 63,806 to 91,758. The average number of SNPs observed in ZH68 plants was the highest (89,544), while that of ZH13 plants was the lowest (81,152). After removing non-polymorphic and missing SNPs, 21,415 polymorphic SNPs were discovered and distributed in all the 20 chromosomes of soybean (Table S2). The lowest number of SNPs was observed in chromosome 11, and the highest number was observed in chromosome 1 (Figure 1c). The average distance between two adjacent SNPs was 33,497 bp, and 97.2% of the SNPs were located in gaps below 150 kb (Figure 1d). The 21,415 polymorphic SNPs were used for the assessment of the uniformity of the five soybean cultivars.

**Figure 2:**
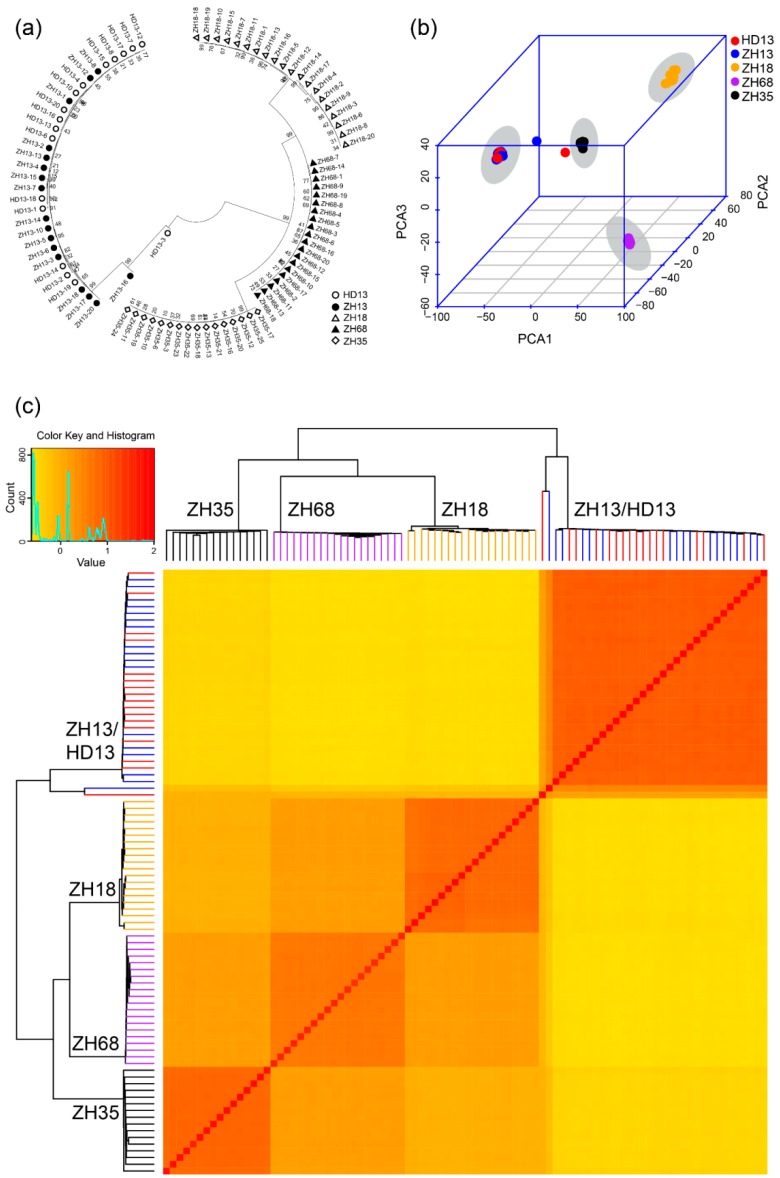
Identification of cultivar distinctness of five soybean cultivars based on the SNPs discovered by SLAF-seq. (**a**) The phylogenetic analysis. White circles represent *cv.* Hedou 13 (HD13), black dots represent *cv.* Zhonghuang 13 (ZH13), white triangles represent *cv.* Zhonghuang 18 (ZH18), black triangles represent *cv.* Zhonghuang 68 (ZH68), white diamonds represent *cv.* Zhonghuang 35 (ZH35); (**b**) The principal component analysis. Red: HD13, blue: ZH13, orange: ZH18, purple: ZH68, black: ZH35. The grey shadow indicates the cluster of the cultivar; (**c**) Heatmap and dendrogram of a stepwise kinship matrix. Colors indicate the cultivars: Red: HD13, blue: ZH13, orange: ZH18, purple: ZH68, black: ZH35.

We applied three methods to analyze the genomic variation of the individual plants to assess the distinctness and uniformity of the five cultivars. Firstly, the Neighbor-Joining phylogenetic tree was constructed for all individual plants using the SNPs and two major branches were generated. One branch comprised of ZH13 and HD13 plants, while the other branch comprised of ZH18, ZH68 and ZH35 plants (Figure 2a). The plants from all the five cultivars were clustered into four groups. The plants from the same cultivar showed less genetic distance and were clustered into the same group. The clusters, ZH18 and ZH68, have closer genetic distance than the other clusters. ZH13 and HD13 plants showed no clear distinction (Figure 2a). Two plants, ZH13-16 and HD13-3, were observed to have farther genetic distance than the other plants of the same cluster and were considered as off-type plants. The pairwise patristic distances of the plants from the same cultivars were closer than those from different cultivars. Further distances between off-type plants and the other plants of its kind were observed (Table 2 and Table S3). Secondly, PCA was performed to investigate the genomic differences of these plants. The first principal component explained 68.8% of the variance observed, while the first two and three principal components explained 85.3% and 94.2% of the observed variances, respectively (Figure 2b). From the 3D plot generated based on the first three principal components, ZH13 and HD13 plants were grouped together, and the plants from the other varieties were grouped by their respective cultivars (Figure 2b). ZH13-16 and HD13-3 were out of the groups and identified as off-type plants. Finally, the stepwise kinship matrix was calculated based on the SNPs, and all the plants were grouped into four subgroups. Except for ZH13 and HD13, plants from other cultivars were clustered into their corresponding groups (Figure 2c) and ZH13-16 and HD13-3 show more distant kinship than the other ZH13 and HD13 plants. Taken together, the plants from *cv.* ZH35, ZH68 and ZH18 were clearly distinct between cultivars and showed a significant distinction with cultivars. However, plants from ZH13 and HD13 showed no distinction from each other. The uniformity of *cv.* ZH35, ZH68 and ZH18 are good at the genetic level and each of the *cv.* ZH13 and HD13 had an off-type plant. 

### 3.2. Assessment of Distinctness and Uniformity of Soybean Cultivars based on SSR Markers

**Figure 3:**
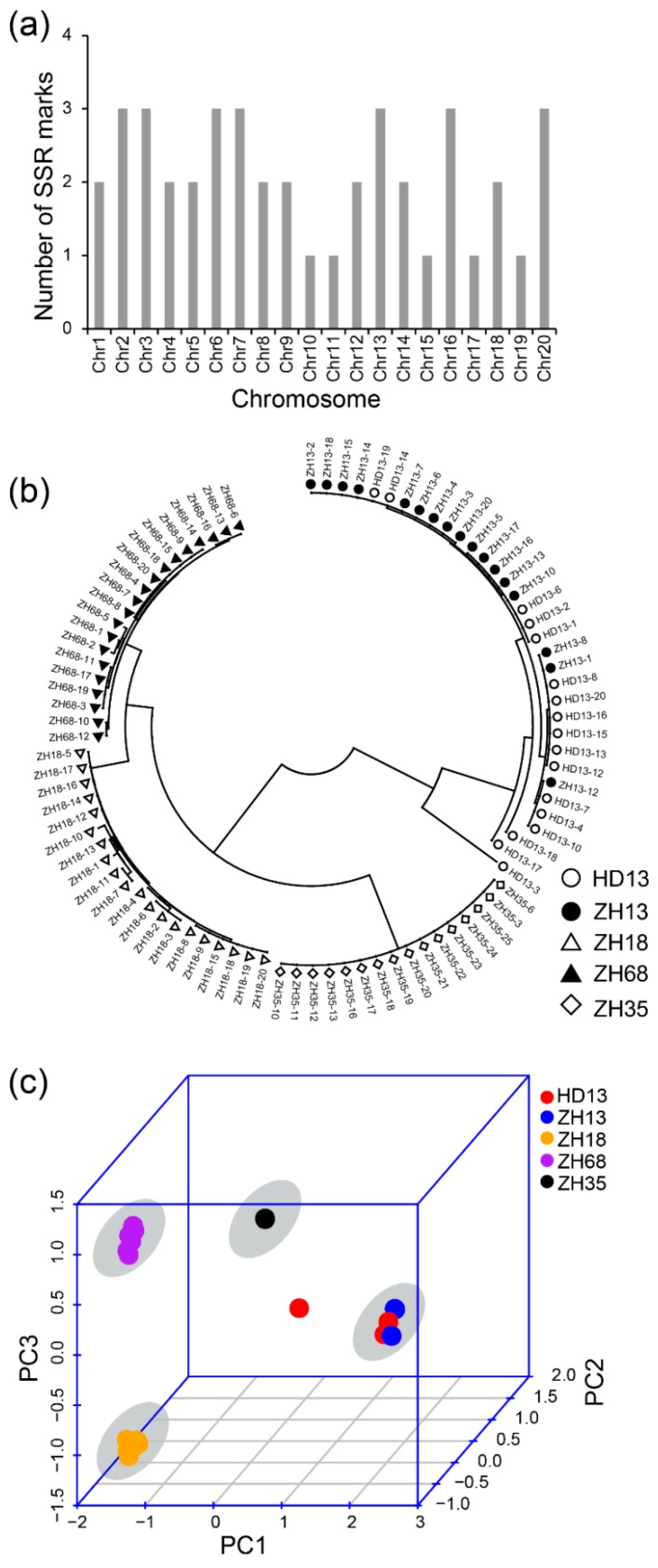
Identification of five soybean cultivars based on the polymorphism of SSR markers. (**a**) The distribution of SSR markers in soybean chromosomes; (**b**) The phylogenetic tree of all soybean plants. White circles represent *cv.* Hedou 13 (HD13), black dots represent *cv.* Zhonghuang 13 (ZH13), white triangles represent *cv.* Zhonghuang 18 (ZH18), black triangles represent *cv.* Zhonghuang 68 (ZH68), white diamonds represent *cv.* Zhonghuang 35 (ZH35); (**c**) The principal component analysis. Red: HD13, blue: ZH13, orange: ZH18, purple: ZH68, black: ZH35. The grey circle indicates the cluster of the cultivar.

As SSR markers are the most commonly used molecular markers for the assessment of uniformity of cultivars, we compared the effect of assessment of uniformity of cultivars using the methods based on SLAF-seq and SSR markers. We used 42 SSR markers that have been previously reported for distinguishing soybean cultivars and evaluating the purity of soybean seeds. There was at least one SSR marker distributed on each chromosome (Figure 3a). The result of the phylogenetic analysis showed four major groups (Figure 3b). The plants of *cv.* ZH13 and HD13 showed no distinction and were clustered in the same group, namely the ZH13/HD13 cluster. Similar plants of the other cultivars were clustered and the clusters were named by their cultivar name. In the ZH13/HD13 cluster, the HD13-3 plant was somewhat different in their clustering from the other plants and considered as off-type plants. The PCA was performed using the genetic distance calculated from SSR markers. The first principal component was able to explain 62.4% of the variance. The first two components explained 75.6% of the variance and the first three components explained 90.3% of the variance. From the PCA 3D plot, all the plants were clustered into four different clusters (Figure 3c). ZH13 and HD13 plants showed no distinction and were located in the same cluster. HD13-3 plant was far from the ZH13/HD13 cluster, which is consistent with the result of the phylogenetic analysis. However, ZH13-16 was not identified as an off-type plant in the SSR results. Taken together, both SLAF-seq and SSR marker methods are able to distinguish the cultivars and identify HD13-3 as an off-type plant; nevertheless, the SSR method did not discover the off-type plant ZH13-16.

### 3.3. The SNP Number Affects Efficiency of Assessment of Distinctness and Uniformity of Cultivars 

**Figure 4:**
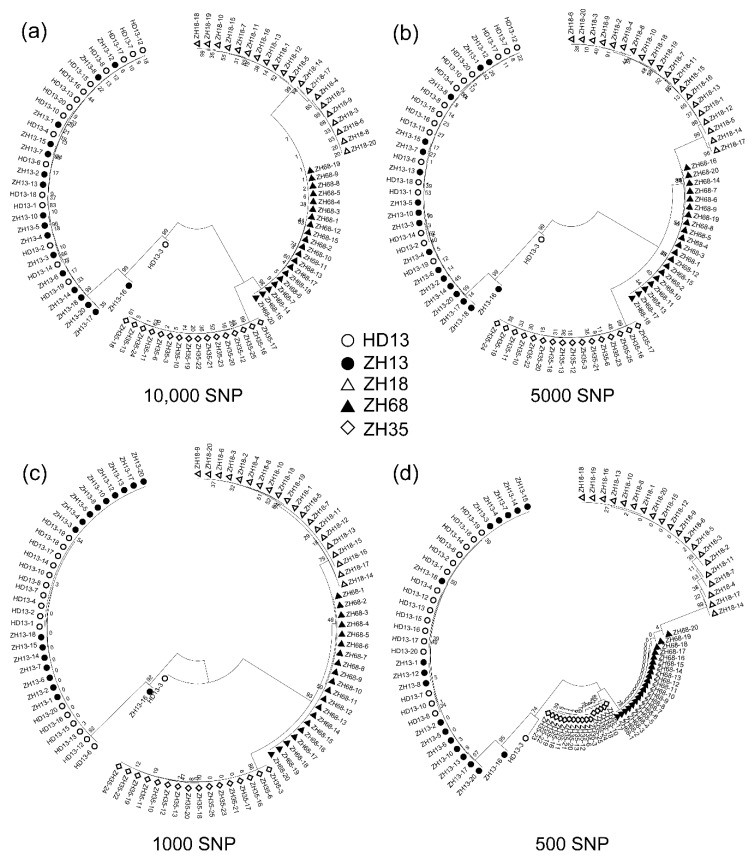
The phylogenetic analysis of the five soybean cultivars using the decreasing number of SNPs randomly picked from all the SNPs identified by SLAF-seq. (**a**) 10,000 SNPs; (**b**) 5000 SNPs; (**c**) 1000 SNPs; (**d**) 500 SNPs.

The density of molecular markers directly impacts the assessment of the distinctness and uniformity of cultivars. The 21,415 polymorphic SNPs discovered were used to construct a detailed fingerprint for the five soybean cultivars to assess the distinctness and uniformity of the five cultivars. The number of SNPs called from SLAF-seq normally depends on the restriction enzyme selection, sequencing depth, and the genomic variation of cultivars. The increase in similarity level of cultivars will decrease the amount of identified SNPs, and thereby the efficiency of assessment of distinctness and uniformity. Based on this reason, we decreased the numbers of SNPs randomly to simulate the estimation of distinctness and uniformity of the cultivars with high similarities. We randomly selected 10,000, 5000, 1000 and 500 SNPs from the 21,415 SNPs using R project. The selected SNPs were used for the construction of the Neighbor-Joining phylogenetic tree to evaluate the genetic variation of individual plants using three replications. We found the number of SNPs indeed affected the structure of the phylogenetic tree, while the plants from the same cultivar still clustered into their corresponding cluster. The phylogenetic trees constructed by 10,000 and 5000 SNPs showed a high similarity of structure compared to those constructed by 21,415 SNPs (Figure 4a,b). With the decreasing SNP number, the structure of the phylogenetic tree changed for the genetic distances of ZH35, ZH18 and ZH68 groups (Figure 4c,d). When the SNP number was decreased to 500, the genetic distance between ZH35 and ZH68 branches declined to the shortest (Figure 4d), suggesting that this SNP number may be a threshold for the effective construction of the phylogenetic tree. However, the distinction between *cv.* ZH13, ZH35, ZH18 and ZH68 was still reliable when the number of SNP was decreased to 500. 

The off-type plants, ZH13-16 and HD13-3, were able to be identified in all the conditions. ZH13-16 was located in the branch containing ZH13 and HD13 in all the conditions. HD13-3 was grouped in the same branch containing ZH13 and HD13 plants in the phylogenetic tree constructed by 10,000, 5000 and 500 SNPs, but was grouped into the branch containing ZH18, ZH68 and ZH35. Nevertheless, in the phylogenetic tree only constructed by 1000 SNPs, HD13-3 was discriminated as an off-type plant in all of the conditions. The result obtained showed that the assessment of distinctness and uniformity of cultivars using SLAF-seq technology is very robust and reliable even with a low number of SNPs. 

### 3.4. Assessment of Genetic Purity for Soybean Cultivars Using SLAF-seq

The genetic purity of each cultivar was analyzed based on SNP markers discovered by SLAF-seq, which provides more informative features than SSR markers. Here, we assessed the purity of each cultivar by the ratio of homozygous loci to all the loci discovered by the SLAF-seq approach (Table 3). All the cultivars possessed a high ratio of homozygous loci ranging from 91.89% to 93.96%. Plants from ZH68 had the highest purity level from 91.36% to 96.25%, and plants from ZH18 had the lowest purity level ranging from 91.38% to 93.19%. Intriguingly, some loci always retain the same heterozygous status in the individual plants within the cultivar. As shown in Table 4, the number of heterozygous loci ranged from 36 in ZH68 to 50 in ZH18. There was the presence of some heterozygous loci among the plants from different cultivars. The loci position of 2,540,914 in chromosome 1 was heterozygous in all the cultivars except ZH35. As the plants tested here were randomly selected, the probability of maintaining heterozygosity at the same locus in tens of plants should be extremely low, which implies that this phenomenon may not be accidental.

### 3.5. The Application of SLAF-seq Technology for the Assessment of the Distinctness in 150 Soybean Cultivars

**Figure 5:**
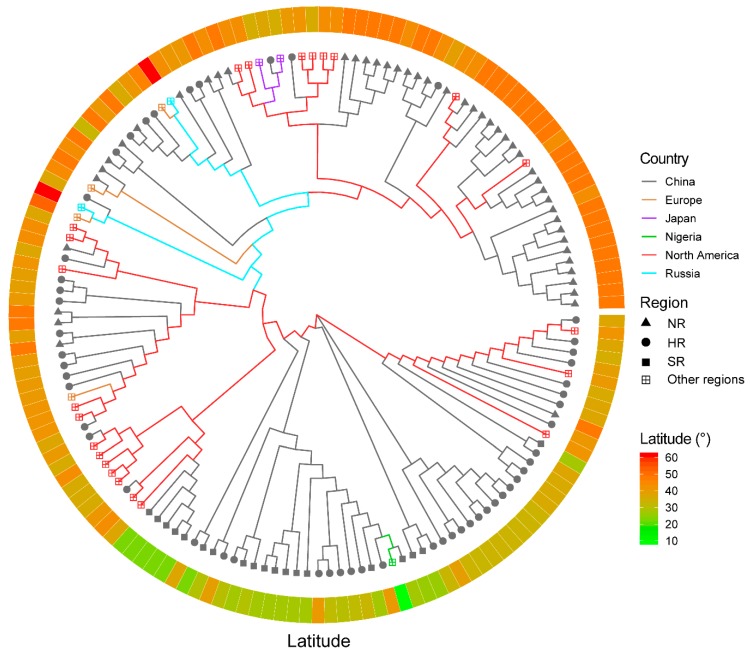
The phylogenetic tree for 150 soybean cultivars. The branch colours represent the countries of origin. The cultivation regions are represented by different symbols of the branches: the solid triangle represents Northern Region of China (NR), the solid circle represents Huanghuaihai Region of China (HR), the solid square represents Southern Region (SR) of China and the square with a cross inside represents other regions (the regions are out of China). The latitudes of the origins of cultivars are indicated by the ring in the outer layer, and the diverse colours represent the latitude degrees (°).

To validate the reliability of the SLAF-seq method in the assessment of soybean cultivars, the SLAF-seq data of 150 soybean cultivars were used to assess the effect of SLAF-seq on differentiating soybean cultivars. The dataset consists of 36,976 SNPs and was used for the further assessment of the SLAF-seq approach for cultivar distinction. The neighbor-joint phylogenetic tree was constructed (Figure 5 and Table S4) and the soybean cultivars with close latitudes were clustered together. The cultivars from Europe (other European countries apart from Russia), Japan, Nigeria and Russia were clearly distinct. The European and Russian cultivars were more closely related. The pairwise patristic distances were calculated for the soybean cultivars (Table S4) and the closest patristic distance (0.105) was found between the cultivars Harosoy and Amsoy and the largest distance (0.508) was found between cultivars TF8 and Jheidou. The results showed that the 36,976 SNP markers were able to outline the distinction among the 150 soybean cultivars, which shows the robustness of SLAF-seq technology. 

## 4. Discussion

The identification of cultivars plays an important role in the modern agriculture industry. Morphological and genetic markers have been applied to define and distinguish cultivars. In recent years, DUS testing based on morphological descriptors is compulsory in the process of registering new cultivars, and DUS testing based on molecular markers is optional. However, some cultivars have narrow genetic backgrounds, which may lead to a lack of proper identification index using morphological descriptors only. Therefore, molecular markers are needed to distinguish between such similar cultivars. Among all the molecular markers, SNP—which is the most abundant in the genome—can be identified in a high-throughput scale and is considered as an ideal molecular marker. In this study, we aimed to establish an effective and easy molecular method for DUS testing in soybean by using SLAF-seq for the high-throughput discovery of SNPs.

SNPs have more abundant and stable genomic variations than other molecular markers, and with the development of SNP identification progresses, more and more studies have been discussing the possibility of applying SNP markers in DUS testing of important crops, such as in wheat, maize, barley and alfalfa [4,44,45,46]. Here, firstly, we applied SLAF-seq to discover SNPs for the assessment of the distinctness of five soybean cultivars after which we further tested the approach on 150 cultivars using SNP data from a GWAS study [6]. In the analysis of the five soybean cultivars, the phylogenetic analysis shows that all cultivars were able to be clearly distinguished. The genetic distance between the two closest varieties was 0.105, which was much further than the genetic distance of the plants from the same cultivars (0.002 ~ 0.052, Table 2), indicating that SLAF-seq has the ability to distinguish between different varieties. Additionally, two direct relatives—*cv.* ZH18 and ZH68—were able to be distinguished by SLAF-seq. Cultivar ZH68 was developed by ICS, CAAS through the crossing of *cv.* ZH18 and Karikoi-434. Although the two cultivars have high similarity in their genetic background (Figure 1b and Figure 3a), they were still able to be distinguished completely using SLAF seq. However, *cv.* ZH13 and HD13, which share a common parent *cv.* Yudou 8, were not able to be distinguished by the phylogenetic tree, PCA, or kinship analysis using both SSR and SNP markers (Figure 2 and Figure 3). The SNPs identified in this study were distributed in all the 20 chromosomes of soybean, and the distance between adjacent markers of most SNPs was below 150 kb. The density of SNP markers was far more than of any of the conventional markers; however, no genetic difference was discovered between *cv.* ZH13 and HD13. Therefore, our results suggest that *cv.* ZH13 and HD13 possibly belong to the same cultivar. 

The number of cultivars with high similarity has a greater impact on the identification of varieties. Therefore, it is suggested to set thresholds for differences within and between cultivars by increasing the number of repeats per variety when the cultivars with high similarity were subjected to being identified. Additionally, SLAF-seq is able to provide more precise fingerprints for cultivars and leads to increasing consideration in lawsuits based upon cultivar infringement.

Although the sequencing methods based on both SLAF-seq and SSR markers showed clear cultivar distinction, they had different effects in the assessment of the cultivar uniformity. The SLAF-seq method showed that ZH13-6 and HD13-3 were off-type plants and the SSR method showed that HD13-3 was off-type plants. Because the marker density of the SLAF-sequencing method was much higher than that of the SSR method, the off-type plants identified by the SLAF-sequencing method may have an extensive difference at the genome-wide level. The SSR method was limited by the marker number, and the detected SSR markers may have a variant in the off-type plants, leading to misjudgment. This misclassification may also be attributable to the possibility of error in alignment because of the low number of SSR markers. The results indicated that the SLAF-seq method was able to show more distinction among the cultivars, even for cultivars with close genetic backgrounds.

The amount of SNPs discovered by SLAF sequencing depends on many factors, such as restriction enzyme selection, depth of sequencing, and genomic variation among the tested cultivars. Previously, SLAF-seq was used in GWAS for soybean cyst nematode resistance in soybean, which led to the identification of 36,976 and 33,194 SNPs from 440 and 200 diverse soybean accessions, respectively [27,47]. Both of the two types of research used *Mse*I and *Hae*III and collected fragments in size of 300–500 bp for sequencing, and the number of SNPs is about 10% more in the 440 accessions than the 200 accessions. In a recombinant inbred population, *EcoR*I and *Mse*I were used for the construction of SLAF and 116,216 SLAFs were discovered, of which 9948 SNPs were polymorphic and were used for the construction of a high-density genetic map in soybean [26]. In the present study, even with the low number of cultivars, we were able to identify 21,415 polymorphic SNPs which were in close comparison to the above studies. This shows that the number of SNPs is likely to not be affected by the number of accessions, but by other factors such as restriction enzymes, genomic variation and depth of sequencing. Furthermore, in order to investigate the effect of decreasing the number of SNPs on the efficiency of assessment of cultivar distinctness and uniformity, we reduced the number of SNPs to 10,000, 5000, 1000 and 500 and simulated the situation that the cultivars with high similarity were identified. With the decrease in the number of SNPs up to 500, the cultivars still showed clear distinction and off-type plants were clearly differentiated. This further indicates the robustness of the SLAF-seq method in the assessment of cultivar distinctness and uniformity.

The purity level reflects the ability to maintain the stability of cultivar characteristics, and usually, every percentage of purity of soybean cultivars resulted in the 450 kg yield loss per hectare [32]. Here, the proportion of homozygous loci was applied to access the purity level of five cultivars based on the SLAF-seq approach. The purity level of each cultivar was above 90%. Most of the approved soybean cultivars are high-generation strains developed through at least six consecutive selfings. Therefore, in theory, the homozygous ratio should be over 90%, which shows an agreement with our result. ZH13 is an elite soybean cultivar with high yield and wide adaptability and has popularized over 6.7 million hectares of land as of 2018 in China. The high purity of cultivar ZH13 contributed to the retention of its excellent characteristics, which ensures its cultivation in large areas for about 20 years. Although the cultivars used in this study had above 90% level of purity, the individual plants from each cultivar exhibited different purities. For example, ZH68 has the widest range of purities from 91.36%–96.26% in the tested cultivars. To obtain higher purified cultivars, further selection should be done to purify the genetic background of cultivars. Additionally, we also found an interesting phenomenon that some loci exhibit the same heterozygous status in all the plants within cultivar and among various cultivars, despite the fact that these cultivars have been cultivated for more than 15 years. This phenomenon has been observed by the previous researches in soybean and Arabidopsis [48,49]. To some extent, the stable heterozygous loci presented in all lines may reflect the high purity and stability of these cultivars as well as the robustness of our approach.

To further validate the applicability of the SLAF-seq approach for distinctness testing, we used SNP information of 150 soybean cultivars with different origins [6]. The phylogenetic tree analysis showed a distinction among the cultivars based on their different origins. The cultivars from China showed a very good differentiation when compared to the North American cultivars. This may be attributed to the exchange of genetic materials between both countries, and the same cultivar may have been recorded with different origins because sometimes the germplasms were not always accompanied by the proper documentation. The cultivars originating from China were also distinguished into their respective regions (HR, NR and SR). The cultivars from SR in China were clearly distinct, while the majority of the accessions from HR and NR also showed clear differentiation, with few cultivars interspersed in both regions. This may also be attributed to an error in the documentation of cultivar information. In a DNA fingerprinting study for the identification of pineapple germplasms using SNP markers [40], they also observed genetic redundancy and the erroneous documentation of germplasms which led to some misclassifications of some germplasms. Therefore, proper documentation is needed in germplasm collections in order to reduce genetic redundancy and misclassification, and such misclassification can only be detected through the application of a molecular approach as demonstrated in the present study. Consistent with the results obtained from the five cultivars, the SLAF-seq approach was demonstrated to be a reliable technique for distinctness testing among soybean cultivars.

The cost involved in the application of technology is very important and should be considered before deploying such technology. In the current study, the costs of SLAF-seq and SSR methods were roughly 67 RMB ($9.66) and 48 RMB ($6.92) per sample, respectively. Although the costs of SLAF-seq were higher than the SSR method, SLAF-seq was able to provide more accurate and abundant information than the SSR method which makes it worthwhile. Moreover, with the development of SLAF-seq, its cost will be gradually decreased in the future research.In general, SLAF-seq has a promising application in DUS testing.

## 5. Conclusions

Taken together, the SLAF-seq approach can be reliably used to complement existing DUS testing based on morphological descriptors in soybean, and this approach has proven to be an accurate method compared to other methods. Our results suggest that the SLAF-seq approach is robust in distinguishing different soybean cultivars and also accurate in assessing the uniformity and purity of soybean cultivars. 

## Figures and Tables

**Table 1:** The pedigrees of the five cultivars.

Cultivar	Breeder	Release Year	Parents	Features
Zhonghuang 13	ICS, CAAS ^1^	2001	Yudou8 × Zhongzuo90052-76	High yield, wide adaptability, disease resistance, lodging resistance, high protein
Zhonghuang 35	ICS, CAAS ^1^	2007	Zhongzuo9943 × Zheng76062	High yield, lodging resistance, high oil
Zhonghuang 18	ICS, CAAS ^1^	2001	Zhongpin661 × Century-2	High yield, disease resistance, lodging resistance, high protein
Zhonghuang 68	ICS, CAAS ^1^	2013	Zhonghuang18 × Karikoi-434	High yield, disease resistance, no beany flavour
Hedou 13	HAAS ^2^	2005	Yudou8 × He95-1	High yield, wide adaptability, disease resistance, lodging resistance, high protein

^1^ Institute of Crop Sciences, Chinese Academy of Agricultural Sciences. ^2^ Heze Academy of Agricultural Sciences, Shandong Province, China.

**Table 2:** The pairwise patristic distances of the plants from the same cultivars.

	ZH13	HD13	ZH18	ZH68	ZH35	ZH13/HD13 ^1^	ZH13-16/ZH13 ^2^	HD13-3/HD13 ^3^
Maximum	0.036	0.028	0.052	0.011	0.014	0.035	0.140	0.233
Minimum	0.010	0.006	0.009	0.002	0.006	0.006	0.123	0.221
Average	0.020	0.019	0.024	0.007	0.011	0.021	0.130	0.229

^1^ The pairwise patristic distances between the plants from ZH13 and HD13 without ZH13-16 and HD3-3. ^2^ The pairwise patristic distances between ZH13-16 and the other plants from ZH13. ^3^ The pairwise patristic distances between HD13-3 and the other plants from HD13.

**Table 3:** The purity of five soybean cultivars based on SLAF-seq.

Cultivar	Purity (%)	Min (%)	Max (%)	Std. * (%)
HD13	92.44	91.32	94.58	0.96
ZH13	92.55	91.24	94.41	1.02
ZH18	91.89	91.38	93.19	0.44
ZH68	93.36	91.36	96.25	1.09
ZH35	92.53	92.32	95.50	1.12

* Std. represented for standard deviation.

**Table 4:** The number of same heterozygous loci between two different soybean cultivars based on SLAF-seq.

Cultivar	HD13	ZH13	ZH18	ZH68	ZH35
HD13	38				
ZH13	21	48			
ZH18	7	8	50		
ZH68	3	10	24	36	
ZH35	7	5	12	16	46

## Supplementary Materials

The following are available online at https://www.mdpi.com/2073-4425/11/2/175/s1, Table S1: The primer information of SSR markers; Table S2: The characteristics of polymorphic SNPs discovered by SLAF sequencing; Table S3: The pairwise distances of 5 cultivars; Table S4: The pairwise distances of 150 cultivars.

## Abbreviations

Specific-locus amplified fragment	SLAF
distinctness, uniformity and stability	DUS
principal component analysis	PCA
International Union for the Protection of New Varieties of Plants	UPOV
fragment length polymorphisms	RFLP
random amplified polymorphic DNA	RAPD
simple sequence repeat	SSR
inter-simple sequence repeat	ISSR
single nucleotide polymorphism	SNP
amplified fragment length polymorphisms	AFLP
reduced representation library	RRL
